# Patients' experiences of living with and receiving treatment for fibromyalgia syndrome: a qualitative study

**DOI:** 10.1186/1471-2474-10-124

**Published:** 2009-10-07

**Authors:** Heidi K Lempp, Stephani L Hatch, Serene F Carville, Ernest H Choy

**Affiliations:** 1 Academic Rheumatology/NIHR Guy's and St Thomas' Foundation Trust Biomedical Research Centre, King's College London, Weston Education Centre, 10, Cutcombe Road, London, SE5 9RJ, UK; 2 Department of Psychological Medicine, Institute of Psychiatry, King's College London, Weston Education Centre, 10, Cutcombe Road, London, SE5 9RJ, UK; 3 UK Age Research Forum, 207-221 Pentonville Road, London, N1 9UZ, UK; 4 Academic Rheumatology King's College London, Weston Education Centre, 10, Cutcombe Road, London, SE5 9RJ, UK

## Abstract

**Background:**

Fibromyalgia syndrome (FMS) presents a challenge for patients and health care staff across many medical specialities. The aetiology is multi-dimensional, involving somatic, psychological and social factors. Patients' views were obtained to understand their experience of living with this long-term condition, using qualitative interviews.

**Methods:**

12 patients were recruited and stratified by age, gender and ethnicity from one rheumatology outpatient clinic, and a departmental held database of patients diagnosed with FMS.

**Results:**

Patients' accounts of their experience of FMS resonated well with two central concepts: social identity and illness intrusiveness. These suggested three themes for the analytical framework: life before and after diagnosis (e.g. lack of information about FMS, invisibility of FMS); change in health identity (e.g. mental distress, impact on social life) and perceived quality of care (e.g. lack of contact with nurses, attitudes of specialists). The information provided from one male participant did not differ from the female patients, but black and ethnic community patients expressed a degree of suspicion towards the medication prescribed, and the attitudes displayed by some doctors, a finding that has not been previously reported amongst this patient group. Patients expected more consultation time and effective treatment than they received. Subjective experiences and objective physical and emotional changes were non-overlapping. Patients' accounts revealed that their physical, mental and social health was compromised, at times overwhelming and affected their identity.

**Conclusion:**

FMS is a condition that intrudes upon many aspects of patients' lives and is little understood. At the same time, it is a syndrome that evokes uneasiness in health care staff (as current diagnostic criteria are not well supported by objective markers of physiological or biochemical nature, and indeed because of doubt about the existence of the condition) and places great demands on resources in clinical practice. Greater attention needs to be paid to the links between the explanatory models of patients and staff, and most important, to the interrelationship between the complex physical, psychological and social needs of patients with FMS. Taking a less medical but more holistic approach when drawing up new diagnostic criteria for FMS might match better individuals' somatic and psycho-social symptom profile and may result in more effective treatment.

## Background

Fibromyalgia Syndrome (FMS) has been described as 'puzzling' [1], and is one of the most prevalent widespread chronic pain conditions, affecting 0.1% to 3.0% of the general population, and 2% to 16% of patients in all clinical situations [2]. It has been recognised within medicine for over a century, and was identified as a distinct clinical syndrome in the 1970s. However, its status as a medical condition remains contested [3]. FMS has been defined as 'a functional somatic syndrome that cannot be explained as a conventionally defined medical disease' [4]. Recent well-designed neuro-imaging studies have revealed abnormal pain perception [5,6].

Little is understood about its precise pathogenesis, although alterations in peripheral and central neuronal hyper-excitability at spinal or brainstem level [7], altered pain perception [8], and somatisation [9] have all been suggested. The aetiology seems to be complex, involving somatic, psychological and social factors, which form the structure of this paper.

Psychological and social aspects commonly are rated as important by patients and are often neglected [10] or viewed differently by clinicians [11]. One third of patients with FMS complain of emotional problems such as depression or anxiety, which exacerbates their pain sensation [12]. The cause and effect relationship between pain and emotional problems remains unresolved. These symptoms are 'common, frequently persistent and are associated with significant distress, disability and unnecessary expenditure of medical resources' [4].

FMS is rarely reported in children, and most patients are in their 40s and 50s with a strong female preponderance (90%) [8]. The syndrome often has a negative impact on patients' daily life and on their ability to continue with work. Currently offered treatment, such as Amitriptyline, Cyclobenzaprine, physiotherapy, psychological treatment and others is only marginally effective [4].

FMS falls under the category of medically unexplained symptoms, in other words patients complain about physical symptoms that are disproportionate to a particular physical disease [13,14]. Almost any medical speciality seems to have one of these functional syndromes and they are a challenge to clinicians [4,14]. In the UK somatic complaints account for 35% of medical outpatients [15] and 20% in primary care [15,16].

### Clinical aspects of FMS

FMS is characterised by allodynia (pain induced by an innocuous stimuli) and hyperalgesia (an increase response to a painful stimuli). Patients experience diffuse chronic pain that they frequently describe as occurring all over the body. Diagnosis is usually made according to the 1990 American College of Rheumatology criteria [17], which state pain must be present for at least 3 months in all four quadrants of the body, and there must be over 11 out of 18 positive tenderpoints at pre-defined locations on the body, identified by pain obtained by light pressure (4 kg/cm^2^). Importantly, these criteria fail to account for the wide range of symptoms that are commonly associated with FMS. The medical term 'syndrome' of FMS reflects the multitude of vegetative and functional symptoms that patients with FMS frequently present, including sleep disturbance, depression, anxiety, fatigue, morning stiffness, irritable bowel syndrome, headache and migraine. In addition, patients often experience a circuitous route through the health care system [18]. Patients with FMS have symptoms that differ from those of individuals with other chronic pain syndromes, e.g. chronic neck or back pain. They have often localised and increased tenderness at predefined sites. Clinical signs of allodynia are not necessary for the diagnosis of many chronic pain conditions [17].

From a patient perspective, FMS is a problematic, invisible condition that is often challenging to health care professionals. At issue is the psychological versus somatic dichotomy, the moral credibility of patients, and the legitimacy of experience and treatment. All of these challenges may jeopardize the therapeutic relationship [19,20]. Further complicating matters, currently there is no effective treatment for FMS that targets all symptoms, nor any consensus on how to best manage the condition [21]. Treatment recommendations are varied from palliating symptoms [22], a multi-modal approach (e.g. exercises combined with education and psychologically-based interventions) [23] or to use across all functional syndromes one diagnostic category with a multidisciplinary approach, due to similarity in symptoms, epidemiology and response to treatment [4]. Recent treatment guidelines from the US [24] and Europe [25] suggested that the best approach is to treat patients based on their individual somatic and psycho-social symptom profile. In the UK Lord Darzi's recent report[26] emphasized the importance of patients' experiences with the goal for NHS staff to deliver an 'inclusive, high-quality, personal health service'.

### Social aspects of FMS

An informal hierarchy of medical specialities exists within medicine [27]. Diseases that are chronic situated diffusely in the lower parts of the body that cannot be investigated or treated by technologically sophisticated procedures, such as FMS and anxiety, are among the lowest ranked conditions [28]. At a structural level, this hierarchy often saddles patients with a heavily stigmatised health identity. From the start of a patient's career, such a diagnosis may compromise their *identity *and core integrity, especially as the legitimacy of the diagnosis is frequently questioned by medical staff [29]. In addition how individuals make sense of, respond to, cope with, and self-manage their illness is deeply socially embedded, and varies not only according to their health identity, but also to social status, such as socio-economic position, age, gender, ethnicity, religion, and disability [30].

Moreover, the notion of *illness intrusiveness *is defined as illness-induced disruptions to patients' valued activities, lifestyle and interests that compromise their quality of life when living with a long-term illness, within the physical, social and emotional domains [31,32]. Illness intrusiveness both mediates between the circumstances of the illness (e.g. pain, disability, fatigue) and its treatment (e.g. side effects, complications) and impacts on person's state of health (e.g. discomfort, contentment).

Within this wider clinical, social and NHS policy context, this study reports the subjective experiences of patients, from diverse ethnic backgrounds, who live with FMS with specific emphasis on how this long-term condition influences their everyday lives, and their contact with primary and secondary care.

This study is part of a collaborative project supported by experts of the European League Against Rheumatism Taskforce. Its aim is to review the current diagnostic criteria of FMS, and thereby to improve the standard of healthcare currently provided. Firstly, the project will start with the concept of FMS as a syndrome of chronic unexplained diffuse pain as a broader baseline description than that used by the 1990 American College of Rheumatology criteria [17]. Secondly, it will identify characteristics of patients, based on their personal views that may be used to classify them into different subgroups in relation to differences in aetiology, pathogenesis and response to possible treatments. Thirdly, three systematic reviews will be conducted of diagnostic criteria, symptoms and instruments used for the evaluation of any of the criteria of FMS or unspecific pain and associated symptoms. Finally evidence will be synthesised through a Delphi method.

## Method

### Recruitment of patients

A purposive sample of 21 patients with FMS was approached whilst at their visit to a rheumatology outpatient clinic run by medical and nursing staff. Patients were not frequent attendees in the rheumatology clinic studied. Therefore additional patients who had previously expressed an interest in participating in FMS research were randomly selected from a departmentally held patient database. They were selected by age, gender and ethnicity. Patients were invited to participate in accordance with the following inclusion criteria: FMS diagnosed by an experienced clinician more than one year ago, over the age of 18 years, able to communicate effectively in English, and with no significant learning, hearing or communication difficulties. The exclusion criteria were: patients that were seriously unwell.

### Interview conduct

Almost all (11/12) interviews took place at the Medical School as patients expressed preference to combine the clinic appointment with their study participation. One patient who was unable to travel was interviewed at home at her request. All interviews were recorded and lasted between one and two hours. Recruitment of new participants discontinued when no new key themes emerged [33]. The researcher used a semi-structured interview schedule based on recent literature [3] and a previous study involving patients with rheumatoid arthritis [10]. Having been piloted for relevance and comprehensiveness of the topics covered, the final interview guide consisted of four main areas: (i) experience of onset of illness; (ii) development of FMS following diagnosis; (iii) the impact on patients' lives, work and family; and (iv) their expectations and experiences when seeking medical help in primary and secondary care. The interviewer's nursing background and extensive clinical knowledge gave her insight into participants' illness experiences. This helped to develop close rapport with patients and in return they provided rich and detailed information. Local Ethics Committee and Research and Development approvals were obtained.

### Data analysis

The interviews were all transcribed verbatim, coded and analysed (content and discourse analyses) with the help of qualitative computer software (NVivo) [34], including simple counting [35]. The data was examined primarily through content analysis (*what *participants' stated). Discourse analysis (*how *patients described their experiences) was also employed [36,37], for example how interviewees described how their self-confidence in private and public gradually waned over time. In this way language was not only viewed as a means of transmitting information or stories, but also as a medium from which additional knowledge could be obtained. This combined application of content and discourse analysis has been endorsed by other researchers [38].

Themes were identified by independently and repeatedly reading the interview text and then formulating an initial coding scheme by one author (HKL). Related codes were used to generate representative themes that emerged from the transcriptions. Dozens of initial codes and related themes were discussed and then cross-checked with the external qualitative researcher (SLH). For this paper, we describe each 'theme' (e.g. the lack of information patients received about FMS following their diagnosis), state how many interviewees discussed the theme (e.g. 11/12) and provide an illustrative account. The single counting procedure [35] is intended to give an indication of the prevalence of the themes in the data, and should not be understood as a basis for statistical generalisation to the population.

Following discussion we further refined the codes by constant comparison within and between the codes to ensure they reflected the data. We then drew up a map to help us to make links between the different themes, which we then grouped into the overall themes. We reached a consensus that three topics constituted our analytical framework under which the data will be presented: (i) life before and after diagnosis; (ii) change in health identity [39,40]; and (iii) perceived quality of care. Moreover, through further discussion we agreed that the significance of the findings under these three headings became further strengthened by linking them to two sociological concepts of social identity and illness intrusiveness. They provided context and served to confirm the relevance and resonance for the key findings of our study.

The bold text within the accounts was included to reflect when interviewees in their talk emphasised points by raising their voice or slowing down their speech.

## Results

From the 21 patient approached, twelve agreed to take part in the study, (11 female, 1 male). Seven were contacted during their rheumatology outpatient clinic visits and five through a departmentally held database of patients with FMS. Of the nine participants who declined, two were male and seven female; all had similar socio-demographic backgrounds to the interviewees, although the mean age was slightly younger (41 years vs. 49 years for participants, see Table 1).

**Table 1:** Socio-demographic characteristics of 12 participants

**Gender**	female 11, male 1
**Disease duration**	Range: 5 months - 11 years Mean 3 years
**Mean age of patients**	range: 20-69 years Mean: 49 years
**Ethnicity (self-described)**	Black 1; Black African 1; Black British1; British/English/White 6; Lebanese 1; New Zealand/British 1; Peruvian 1
**Family status**	married 5, married living apart 1; divorced 1; single 4; not specified 1
**Place of birth (country)**	UK 8, outside Europe 4
**Family dependents**	4/12 patients (young children 3-18 years) 2/12 patients (caring for their older mothers)
**Employment situation**	full-time work 3; temporary employment 1; retired on medical grounds 1; retired 2; unemployed 5.
**Registered disabled**	7/12 patients

### Life before and after diagnosis

All patients (12/12) described at least three factors that they believed had contributed to the development of FMS. Four categories emerged out of the diverse accounts: (i) physical ailments with long-term pain (12/12), e.g. 'growing pain', accidents, surgical interventions, (ii) family history (9/12), e.g. diagnosis of arthritis or generalised aches and pains by other family members; (iii) specific personality type (6/12), e.g. working all the time, involvement in competitive sport; (iv) mental stress (7/12), e.g. job loss, bereavement, unhappy marriages.

'I used to go to work as a young woman; I worked up in town and there was always an element of pain, but not as I am now. ... I am in this marriage that isn't happy, it is a dreadful marriage, after 48 years nearly I think it's beginning to take its toll'. (Patient 12)

The majority of participants (11/12) had their diagnosis confirmed by a rheumatologist or orthopaedist. Diagnosis was made by medical staff after enquiring about symptoms, and by applying pressure to specific body tenderpoints that can produce severe pain (7/12). Most patients (8/12) had been referred by their GPs, or received the diagnosis during their routine rheumatology outpatient visits.

'It was in XX hospital and he [rheumatologist] did an examination of pressing on certain parts of my anatomy - shoulders, back, body, wherever and it was quite painful. It wasn't just pressure it was pain. And after the diagnosis he [rheumatologist] said to me I was suffering with this fibromyalgia'. (Patient 12)

Almost all patients (11/12) critically remarked that very little or no information was given to them following their FMS diagnosis. This required the interviewees to be proactive in seeking information from other sources, such as the internet, leaflets, self-help groups or books. This lack of medical understanding about FMS made explanations about the condition to patients, family, friends and colleagues difficult. A few (3/12) stated they did not understand the diagnosis at all.

'For me it [FMS] just feels like an all over body... like a disease, I don't know, I can't really describe it. All I know is that it just, well it takes its toll, it doesn't just affect one part, and it affects your whole body'. (Patient 4)

The invisibility of FMS was an additional difficulty facing most patients (9/12). They reported an incongruity between how they look to others 'from the outside' that usually was not mirrored by how they felt 'inside'.

'Oh I'll put on my lipstick and get ready for work, dress smart and they'll [colleagues] say: "oh you look lovely, you look nice", but inside I feel really, really awful'. (Patient 10)

All described some mixture of emotions, such as relief (5/12), ambivalence (3/12), or negative reactions (4/12) following the formal confirmation of their FMS diagnosis.

'....FMS just feels sometimes engaging with life from behind it, a big screen of glass where you can **see **things going around you and you think: I want to be part of that, I am going to want to be part of **that**. And to do that, you have to kind of **smash **the glass through, but obviously you'll get hurt, there is going to be a bit of pain or consequences if you do sort of smash through'. (Patient 6)

The reactions of family members, friends and colleagues were not straightforward, and seemed to reveal and amplify the quality of pre-existing relationships that had developed over time. The following contrasting accounts (6/12 positive, 6/12 unsympathetic) illustrate two common responses:

'My family are so supportive, a small knit family but very supportive, everybody helps'. (Patient 11)

'My family could not understand it. It was something alien to them. My father was totally oblivious to it... he just could not cope with it [diagnosis] at all'. (Patient 1)

### Change in health identity

Patients' health identity appeared to gradually transform following the diagnosis and the impact and intrusiveness of the illness into their lives became increasingly apparent. FMS affected patients physically, socially and mentally and seemed to progressively undermine their self-confidence and sense of self.

Almost all (11/12) commented about their inability to rely on their body in ways that had not been obvious before the diagnosis. The most frequently used phrases were 'very frustrating' and 'scary' or 'frightening'. In addition, their self-confidence gradually became undermined in public, at work and in social situations, which in part was attributed to the unpredictability of the illness course.

'It is frustrating, very frustrating, because you are really not sure how you are going to be sometimes from one day to the next, so that is difficult'... (Patient 3)

'I felt in a space of a year being amazingly competent and confident and feeling on top of things to actually the complete opposite, and not understanding why and not understanding how it happened'. (Patient 6)

### Physical problems

The descriptions of patients' pain experiences (12/12) engendered the most detailed and lengthy data. Indeed, 'pain' was the most frequently used key word throughout the interviews. In addition to reports of severe pain, hurting in the joints and muscles was amplified by a wide range of additional sensations such as stiffness, spasms, or burning.

'... it feels as though I am walking with a big cross nailed on my back'. (Patient 12)

'Pain is everywhere... sometimes especially my left arm, I feel like cutting it off... just taking it raw, no anaesthetic nothing, it must be cut off'. (Patient 5)

What emerged from these detailed reports was a common description of pain that: (i) spread from head to toe, affecting most of their joints and muscles; (ii) took its toll physically and mentally; (iii) triggered behaviour change; and (iv) seemed constantly present. The pain appeared to signify a disruption in that a third of patients (4/12) had no recollection of any pain-free time in their lives since diagnosis, and only a few (2/12) could vaguely remember short periods of living pain free.

In addition to the overwhelming nature of pain, all participants (12/12) complained about the lack of restful sleep for many years that resulted in chronic fatigue for half of them (6/12). One patient mentioned the tiredness as more debilitating than the pain. The suggested causes of insomnia were pain coupled with an uncomfortable stiffness that was difficult to alleviate, particularly during the night. For some (5/12) this lack of sleep had an impact on their behaviour during the day or night, as the following account illustrates:

'Sleeplessness nights ... can you see the black circles under my eyes, because I wake up at 2 o'clock in the morning, then I wake up and feel right awake...and the next day I feel tired, I just want to sleep on but I have to get up and go to work ... I am extremely physically tired ... I am so tired I just can't function as a normal person'. (Patient 9)

Abdominal discomfort was another physical problem that was commonly reported (11/12). The descriptions referred to a range of specific or non-specific conditions, e.g. irritable bowel syndrome, Crohn's disease, and change in bowel habits, which were troublesome and often embarrassing.

Apart from these debilitating symptoms of pain, fatigue and abdominal discomfort, many (9/12) encountered difficulties with mobility, especially when travelling. Most patients (7/12) had access to a car/taxi, but some relied on public transport. Others made use of their Disability Living Allowance and/or family members who provided transportation.

'I use the bus. It is OK with the bus, but I only go to those buses that are not full, so I can sit [down]. I can't stand in the bus from one bus stop to the other, so I wait until the rush hour is over and then I go out'. (Patient 2)

Additional co-morbidities were common (11/12), for example, non-specific symptoms such as frozen shoulder, headaches, clumsiness and menopausal complaints. Patients reported up to 10 co-morbid complaints, covering almost all body systems.

### Mental Distress

Mental and neurological related distress in FMS appeared to affect patients in three ways: in terms of depression/anxiety; cognitive problems; or a lack of co-ordination. The following phrases were articulated by many (11/12) and reflected their degree of distress:

'I was a complete mess'; 'I was feeling suicidal'; 'very, very weepy'; 'mentally and physically shattered'; 'feeling very, very down'; 'I am in a mental cocoon, in a cage, in a bubble'; 'I was at a low point, in a black hole'.

The manifestation of 'feeling low' triggered changes in behaviour, but at the same time, patients described attempts to avoid 'spiralling down' in their mood.

'I get quite depressed, particular as I am getting older, feeling less and less [physically] able. The major thing about it is the moaning aspect actually, I hate it and I keep thinking: moan, moan, and moan. I take regular anti-depressants at night; listen to music, sometimes I pray: "please Lord just take this pain away, I'm just so sick and tired of it" '. (Patient 8)

Distressing symptoms such as an inability to remember facts (5/12) also were brought up. Problems with memory were referred to by some (3/12/) as 'fibro-fog' or 'foggy-brained'. The following is an example of patients' problems with coordination.

'I sat up sometimes and smacked my head into the door. So I now make sure the door [in my flat] is open, because I can't coordinate properly, I just smack into things and half of the time I am not realising it'. (Patient 3)

### Impact of FMS on patients' social life

Patients' social lives also became compromised, including their determination to remain independent. Many (11/12) reported changes in their social life after diagnosis, which was mostly characterised as being less able to go out and enjoy themselves with friends and family. A few patients (2/12) had physical problems that caused embarrassment or isolation, and one chose work ahead of having a social life.

'My social life is very limiting, very limiting... I always need to sit down and it's very difficult going round talking to people and I love people. I am a gregarious person and that is very difficult and so I am tending to lose out a bit'. (Patient 8)

At the time of the interview about half of the patients (5/12) were unemployed due to the physical discomfort and restrictions caused by FMS symptoms and three were retired (3/12). Some (4/12) felt they were able to continue with paid work (see Table 1) despite occasional unsympathetic reactions by colleagues, and over a third (5/12) expressed a sense of isolation in the work place.

'And at work... they [colleagues] do not understand it [FMS]... they sort of forget sometimes.... we are not allowed to leave our seat; we are not allowed to get a drink of water. So I have to ask: "can I have a break?".... I need to be able to move, otherwise I seize up, and that makes me worse for the evening, but my colleagues are not going to see that'. (Patient 3, full time work)

Lack of awareness about FMS seemed to play a role in how patients experienced stigmatisation or discrimination in public. Many (10/12) described encounters when they were viewed as an 'invalid' by friends/colleagues, or they described overhearing remarks about their obesity. Patients often referred to their deliberate choice not to use a stick at work or in public to avoid embarrassment or being asked direct questions about their illness.

'I am not at work on Thursdays, so they [colleagues] say: "why are you not here on Thursdays" and sometimes I don't explain. But sometimes I have to explain; and some people you know have looked at me as if to say: "well you are a disabled person now; you are not a full person any more"... (Patient 9)

In fact, most (7/12) were registered as disabled (see Table 1) and talked about their reactions when enquiring about their entitlement to Disability Allowance. Half (6/12) welcomed their additional income and special parking badge and remarked how these contributed to a better quality of life, e.g. to greater independence, mobility, and comfort, despite the initially complicated bureaucracy. Some (4/12) wanted to delay their application and others (2/12) found the step problematic.

'Oh I so loved my work, it was really exciting... so then... yes it's difficult isn't it [to ask for disability allowance] but on the other hand it is a relief to actually get some help... at least it is helping us [wife and me] out and ...I am really pleased to get disability allowance'. (Patient 8)

Patients' self-determination while living with FMS was an important attribute in their self-management. What emerged from their detailed descriptions was a fine balance between a desire to remain independent as long as possible (11/12), and also wanting and needing support (9/12). Patients found ways of managing their lives by making compromises and by sharing activities with the help of supportive family members and friends (9/12). Only two (2/12) talked about input from a formal care supplier, e.g. Social Services, or state provided household adaptations.

'I can do everything myself, shopping, cooking, cleaning, very, very slowly. I just do small amounts and stop, small amounts and stop'... my daughter comes, she helps me, she is very, very good'. (Patient 1)

Half of the interviewees (6/12) were married and they all revealed ways in which the illness took its toll on their relationships.

'FMS affected my life a lot, it affected my marriage and my marriage broke down [and] my heart. I had a wonderful husband, he was in Nigeria and I was in London...' (Patient 11)

Finally, half of the interviewees (6/12) expressed worries about how they would manage their illness in the near or long-term future.

'I am so scared; my oldest daughter is not going to look after me, who will look after me when I get worse'? (Patient 7)

For some informants (2/12), stress was amplified by additional caregiver responsibilities for dependent family members, e.g. old parents.

### Perceived quality of care

A surprising finding was the patients' lack of regular contact with nurses in both primary and secondary care (11/12). In primary care, the main concern was the perceived lack of time by GPs in their consultations (9/12). The majority (7/12) provided positive comments, e.g. that the GP was sympathetic or supportive, but some (3/12) complained about GPs' (i) unresponsiveness to their needs, and (ii) perceived function that was limited to prescribing medication.

'He [GP] is understanding. He does not have much time that is why I asked to see a rheumatologist. He is so fast, only 5 minutes: **"**and what do you have? More prescription ok, here you are, bye, bye" '. (Patient 2)

In contrast, patients' comments (12/12) about hospital specialist outpatient care focused upon their expectations about: (i) the professional attitudes and behaviour of specialists; and (ii) organisational aspects.

'I'm not happy ... my appointments have been cancelled several times... I sat about two hours in the waiting room and had a 15 minutes appointment and had my medication changed and that was it, that was all... that is not good enough'. (Patient 4)

Another notable finding was reflected in the critical comments made by patients (4/12) who identified themselves as members of black and ethnic communities. They voiced directly and indirectly uneasiness about certain aspects of their care (e.g. being prescribed drugs) and a general mistrust towards health care professionals.

'I am on Amytriptiline to help with my sleep and other tablets... I only take them when it is right for **my body**. I don't like to be taken as a guinea pig. I don't trust staff to deal with me in that way. I am the **only **person who knows what it feels like to be ill, and what is good for my body, I don't like other people to tell me and to control me'. (Patient 7, Black)

Most patients (9/12) were very resourceful in controlling their symptoms through practical and mental distractions, but some (3/12) recalled crisis situations of unbearable and uncontrollable pain. Almost all (10/12) expressed ambivalence in relation to the prescribed medication. They were worried about two particular issues: becoming dependent on medication and that the tablets 'masked' their overwhelming symptoms without curing the underlying problem. These worries seemed to be linked to their own beliefs about treatment and many (11/12) felt that the prescribed drugs were ineffective in relieving their pain and discomfort. Most admitted that they followed their own intuition, rather than medical advice.

'With my back, the more you put pressure on the more you feel the pain. Ibuprofen cools the pain down a little bit, it helps you to move around... the Doctor said I can take Paracetamol... it relieves me a bit from the pain...the one with the codeine [Co-Codamol] I don't take anyway...'(Patient 5)

Apart from medication, many patients (10/12) were offered physiotherapy as part of the treatment package. While a few (4/12) made positive comments, for many (6/12) the word physiotherapy brought up negative memories.

'Don't talk to me about physiotherapy, I hate it! I have tried it. It did nothing for me. I **hate **it! It caused more pain believe it or not. I did not think it would, but it did. I was in agony. And it was only gentle physiotherapy, but I did not like it'. (Patient 11)

Finally, many (9/12) had tried or used alternative medicine/therapy in addition to their prescribed medicine. Patients admitted reluctantly little long-term improvement, apart from applications of heat in different forms (7/12) (e.g. swimming in warm water or the use of a hot water bottle).

## Discussion

These in-depth interviews confirmed the many consequences associated with FMS including severe negative physical, mental, and social impacts. These sometimes became intrusive and overwhelming. FMS is a diagnosis that is contested between health care staff and patients. Participants appeared to be caught between their daily subjective experiences and attempts at self-management, and their frustration having to prove their illness to health care staff who often cannot objectively detect any physical changes. This has also been described by other authors [41,42]. Patients depend upon a medical diagnosis to continue in their 'sick role' and receive some acknowledgment of being unwell by family, friends and work colleagues. This notion has been identified by Parsons [43]. However, some of its aspects, the lack of objective indicators that traditionally contribute to formalising a medical diagnosis and FMS's long-term prognosis is problematic in this context. As a consequence, these unresolved tensions tend to surface for patients during their encounters with health professionals. In fact many long-term conditions now contribute more towards the global burden of disease in high income countries than do acute diseases [44]. Within the long-term condition paradigm, the traditional passive patient role is increasingly redundant [45].

Before discussing the results in detail, there are some strengths and limitations that deserve attention. Many reviews and publications of qualitative studies focused mainly on women [3,46-50], due to the disproportionate prevalence of FMS among females [8,13]. While our study may have benefited from the inclusion of more than one male patient, there were no notable differences by gender with regards to physical and psycho-social experiences. This is consistent with the findings from a study of men with FMS [51] that revealed also parallel findings that pointed towards difficulties in relation to identity and dealing with overwhelming pain. This qualitative study represents the views of a small number of patients from one outpatient clinic and therefore, the findings are not necessarily generalisable. However, the findings are timely and do represent direct insights from the perspective of patients with FMS, and their views and quality of health care provided within the NHS, priorities laid down in a recent report [26]. To our knowledge, only one other paper has been published about the views of black and minority ethnic group patients with FMS [52].

Many of the participants did not work with a mean age of 49 years and over half were registered as disabled. This indicates that our patient group appeared to experience a more 'severe' form of FMS and therefore studies that include people who are cared for by primary care only would be important for future research.

Another consideration is this study's reliance on in-depth interviews as our only method for extrapolating data. This project may have been strengthened with methodological triangulation, by administering questionnaires with validated scales representing some of the aspects of physical and psychosocial functioning [53]. Obtaining the data through focus groups was considered, but we anticipated that patients preferred one to one interviews due to the very private and personal disclosures, that in return provided us with rich information.

### Physical and mental impact of FMS

The two main social concepts of illness intrusiveness [31,32] and social identity [39,40] were strengthened and relevant to our findings. Specific manifestations of FMS did seem to have a detrimental effect upon patients' sense of self. In particular, widespread pain, abdominal discomfort, insomnia, and mood swings all contributed to a changed sense of identity for many patients. These elements were highly inter-related, for example behavioural changes that were triggered by pain in turn resulted in lack of self-confidence. The invisibility of FMS, despite its intrusiveness, presented patients with additional social difficulties [54] and was portrayed by many as a discrepancy between their outside appearance and their inner fragility [48]. The lack of acceptance by significant others of FMS as a 'proper' illness was experienced as deeply problematic [46]. This was compounded by the lack of knowledge about FMS by the patients and the public, and contributed to experiences of stigmatisation and discrimination, as reported in other visible [55] and invisible long term conditions [56,57]. Interestingly, several patients said having a visible marker of their condition, such as a walking stick or a wheelchair, was helpful to gain greater legitimacy and respect. Such obvious helpful signs have also been mentioned by patients with rheumatoid arthritis [58].

Pain and muscle soreness were dominant symptoms and heavily contributed to troubling themes in their lives, as highlighted by other publications [30,46-50,57]. The depictions of chronic pain were powerfully expressed through picturesque talk. This can be interpreted as overstatements and/or tensions that may occur within medical consultations when people perceive their lives as controlled or misunderstood by others [59]. It serves to emphasise the difference between one group to another [60], in this case between the patient with FMS and health care professional. Patients clearly pointed towards the relationships between body and mind and how pain, insomnia, and specific life events were often related to their mental health. Therefore, it was apparent that patients often found it difficult or impossible to separate physical and mental pain [3,61]. Further, lack of restful sleep, abdominal discomfort, and mood disturbances were common features of distress that were frequently reported by patients, but are not included in the 1990 diagnostic criteria [17,62]. Some could not remember a pain-free time since their diagnosis. A recent randomised controlled trial of a sleep quality modifier showed improved mood and fatigue and reduced pain that are an important step in the treatment of patients with FMS [63].

### Social implications of FMS

The debilitating physical and emotional symptoms had socially limiting effects on many patients' lives. For example, going out was less enjoyable and less spontaneous, and transport and mobility became problematic. For some this led to social isolation, in particular when employment was no longer an option and relationships became strained [30,64]. Not all patients with a long-term condition find their social life curtailed or experience loneliness, however, unemployment and difficulties in relationships are common [58].

A determination to remain independent, even while accepting help from others exemplifies the challenge of finding balance of dependence and independence that faces many people with long-term conditions. This is closely linked with notions of respect and dignity through maintaining self-control and self-responsibility [48,57,65]. The uncertainty about the long-term course of FMS made these self-management elements particularly difficult to foresee or plan for the future, which contributed to additional anxiety and stress. Thus, many chose to take life on a 'one day at a time' basis.

### Views on quality of care

Patients' critical view of primary and secondary care is not surprising, given the relative lack of understanding of FMS, and controversy about its legitimacy [4]. GPs tend to see themselves as specialists in long-term conditions, although there is absence of evidence of effective collaboration between primary and secondary care, and comprehensive management for patients with such conditions [66]. The contribution of specialist rheumatology nurses with this patient group recently has been highlighted [67]. The lack of consultation time with doctors was criticised by patients, and this could be complemented by nurse consultations.

Patients' reluctance to take medication as prescribed, mostly in long-term conditions, is well known [68]. In this study, mistrust of prescribed medication particularly was found among patients from black and ethnic communities, as shown elsewhere [69]. They expressed their uneasiness towards prescribed medication especially if they felt that medical staff could not clearly communicate the effects and side effects of the treatment. It is increasingly important for health care practitioners to develop an understanding about potential sensitivity from black and ethnic minority patients about their reluctance to follow advice e.g. medication concordance, change in life style or health behaviour due to past experiences in the health care system with sometimes perceived unfair or unacceptable treatment offered.

## Conclusion

From the point of view of patients, FMS has been portrayed in this study as an overwhelming condition that intrudes upon many aspects of their lives and is little understood by health care professionals, friends, family and the public. At the same time in the medical literature, FMS is described as a syndrome that evokes uneasiness in health care staff (as current diagnostic criteria are not well supported by objective markers of physiological or biochemical nature and indeed because of doubt about the existence of the condition at all) and places great demands on resources in clinical practice. Greater attention needs to be paid to the links between the explanatory models of patients and staff, and most important, to the interrelationship between the complex physical, psychological and social needs of patients with FMS. Taking a less medical but more holistic approach when drawing up new diagnostic criteria for FMS might match better individuals' somatic and psycho-social symptom profile and may result in more effective treatment.

## Competing interests

HKL and EHC: are supported by the NIHR Biomedical Research Centre, Guy's and St. Thomas' Foundation Trust/King's College Hospital Foundation Trust and King's College London.

SLH: is supported by the NIHR Biomedical Research Centre for Mental Health at the Institute of Psychiatry, The South London and Maudsley NHS Foundation Trust and Kings College London.

SFC: has no competing interest.

## Authors' contributions

HKL: designed the study, carried out all interviews, and conducted the data analysis.

SLH: advised and contributed to the data analysis.

SFC: advised on the study design.

EHC: advised on the study design.

All contributed to writing of the paper.

## Pre-publication history

The pre-publication history for this paper can be accessed here:


